# Steeper lateral posterior tibial slope and greater lateral-medial slope asymmetry correlate with greater preoperative pivot-shift in anterior cruciate ligament injury

**DOI:** 10.1186/s40634-022-00556-x

**Published:** 2022-12-07

**Authors:** Kiminari Kataoka, Kanto Nagai, Yuichi Hoshino, Masashi Shimabukuro, Kyohei Nishida, Noriyuki Kanzaki, Takehiko Matsushita, Ryosuke Kuroda

**Affiliations:** Department of Orthopaedic Surgery, Kobe University Graduate School of Medicine, 7-5-1 Kusunoki-Cho, Chuo-Ku, Kobe, Hyogo 650-0017 Japan

**Keywords:** Anterior cruciate ligament, Electromagnetic measurement system, Pivot-shift test, Posterior tibial slope, Quantitative measurement

## Abstract

**Purpose:**

To investigate the association between posterior tibial slope (PTS) and preoperative pivot-shift phenomenon in anterior cruciate ligament (ACL)-injured knees.

**Methods:**

Fifty unilateral ACL-injured patients (mean age: 28.0 ± 11.4 years, 29 males) who underwent ACL reconstruction were retrospectively included. Patients with a history of injury to the ipsilateral knee joint, concomitant ligament injuries with ACL injury, and/or more than one year from injury to surgery, were excluded. Pivot-shift tests were performed preoperatively under general anaesthesia using an electromagnetic measurement system, and tibial acceleration (m/s^2^) during the posterior reduction of the tibia was measured. Medial and lateral PTS (°) were measured respectively using high-resolution CT images taken two weeks after surgery. Lateral-medial slope asymmetry was calculated by subtracting medial PTS from lateral PTS (lateral-medial PTS) and we evaluated the correlation between each PTS parameter (medial PTS, lateral PTS, and lateral-medial slope asymmetry) and tibial acceleration during the pivot-shift test. The level of significance was set at *p* < 0.05.

**Results:**

Medial PTS was 4.9 ± 2.0°, and lateral PTS was 5.2 ± 1.9°. The lateral-medial slope asymmetry was 0.3 ± 1.6° (range: -2.9 to 3.8). Tibial acceleration during the pivot-shift test in the ACL-injured knee was 1.6 ± 0.1 m/s^2^. Preoperative tibial acceleration was positively correlated with lateral PTS (*r* = 0.436, *p* < 0.01), and lateral-medial slope asymmetry (*r* = 0.443, *p* < 0.01), while no significant correlation was found between preoperative tibial acceleration and medial PTS (*r* = 0.06, *p* = 0.70).

**Conclusion:**

Preoperative greater tibial acceleration during the pivot-shift test was associated with steeper lateral PTS and greater lateral-medial slope asymmetry in ACL-injured knees. These findings improve our understanding of anterolateral rotatory knee laxity by linking tibial bony morphology to quantitative measurement of pivot-shift phenomenon. Surgeons should be aware that not only lateral PTS but also lateral-medial slope asymmetry are the factors associated with preoperative pivot-shift.

**Level of Evidence:**

Level IV.

## Introduction

In anterior cruciate ligament (ACL)-injured knees, the cause of the spectrum of anterolateral rotatory knee laxity is multifactorial: the degree or chronicity of the injury to the ACL; injury to secondary knee stabilisers (i.e. lateral meniscus and anterolateral complex); bony morphology (i.e. intercondylar notch width and the shape of the tibial eminence) as well as patient characteristics (i.e. sex and generalised joint laxity) [4, 5, 13, 25]. The pivot-shift test is the most commonly used clinical examination to evaluate anterolateral rotatory knee laxity [22]. The preoperative pivot-shift test has particular clinical importance because greater preoperative pivot-shift has shown to be associated with residual post-operative pivot-shift after ACL reconstruction [33]. Moreover, residual pivot-shift after ACL reconstruction has shown to be associated with symptoms and functional outcomes [2]. Therefore, a better understanding of the pivot-shift phenomenon is considered clinically important.

Morphological factors have been attracting increased attention as important factors for unfavourable knee kinematics and ACL injury in recent years. Especially, steeper posterior tibial slope (PTS) has been recognised as a potential risk factor for ACL injury [7], ACL graft failure [29], and post-operative static anterior tibial translation [19]. In terms of anterolateral rotatory knee laxity, several studies have shown that steeper PTS is associated with a high-grade pivot-shift which was evaluated by subjective grading [3, 4, 31]. Another study has shown that not only lateral PTS but also lateral-medial slope asymmetry were risk factors for concomitant posterolateral meniscal root tears in ACL injuries, suggesting lateral-medial slope asymmetry causes tibiofemoral rotation and increases the load on the posterolateral meniscus root [17]. However, an association between PTS and preoperative pivot-shift has not been fully elucidated, and the evidence of quantitative evaluation of the pivot-shift test was still lacking. Thus, the association between PTS/ lateral-medial slope asymmetry and pivot-shift phenomenon remains unknown.

Recently, the usefulness of quantitative evaluation systems for assessing anterolateral rotatory knee laxity has been reported [10, 21], and an electromagnetic measurement system (EMS) is one of these systems [1, 8, 12, 20, 24]. Therefore, we aimed to investigate the association between PTS/lateral-medial slope asymmetry and preoperative pivot-shift. It was hypothesised that steeper lateral PTS and greater lateral-medial slope asymmetry would be associated with greater tibial acceleration during the pivot-shift test which was measured using the EMS.

## Materials and methods

### Study design and population

The present study was approved by the Institutional Review Board of Kobe University (ID: B190055). Informed consent was obtained from all participants in the present study.

**Fig. 1:**
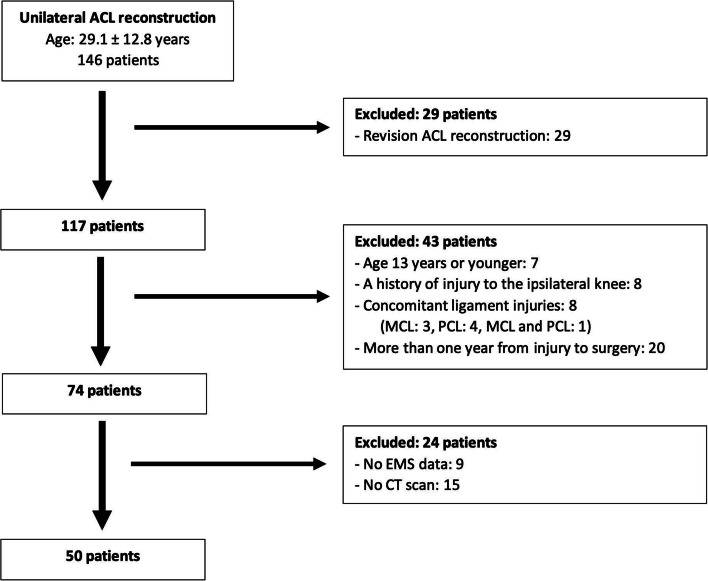
Flowchart of patient enrollment

A retrospective analysis of prospectively collected data was performed. ACL-injured patients who underwent ACL reconstruction at our institution between January 2017 and March 2021 were prospectively enrolled. A total of 146 patients of ACL reconstructions were performed. Revision ACL reconstruction, age at the time of injury was 13 years or younger, patients with a history of injury to the ipsilateral knee joint, concomitant ligament injuries with ACL injury, no data of the pivot-shift test measured by the EMS, no available CT scan, and/or more than one year from injury to surgery, were excluded. As a result, 50 unilateral ACL-injured patients (mean age: 28.0 ± 11.4 years, male/female: 29/21) who underwent ACL reconstruction were included in the present study. A detailed flowchart is shown in Fig. 1. Among these patients, 37 patients with concomitant meniscus injuries (lateral meniscus: 18, medial meniscus: 13, lateral and medial menisci: 6) and 11 patients with concomitant cartilage injuries (medial femoral condyle: 8, lateral tibial plateau: 1, trochlea: 1, lateral femoral condyle and lateral tibial plateau: 1) were included.

### Computed tomography protocol and measurement of PTS

Post-operative CT scans were taken approximately two weeks after ACL reconstruction to evaluate tunnel locations. The knee was scanned from 5 cm proximal to the femoral epicondyle to 5 cm distal to the tibial tubercle using 64-slice MDCT (Aquilion 64; Toshiba Medical Systems, Tokyo, Japan). The slice thickness was 0.5 mm and one slice of the post-operative MDCT image consisted of 512 × 512 voxels. Multi-planar reconstruction was performed to obtain sagittal slices of the knee joint.

**Fig. 2:**
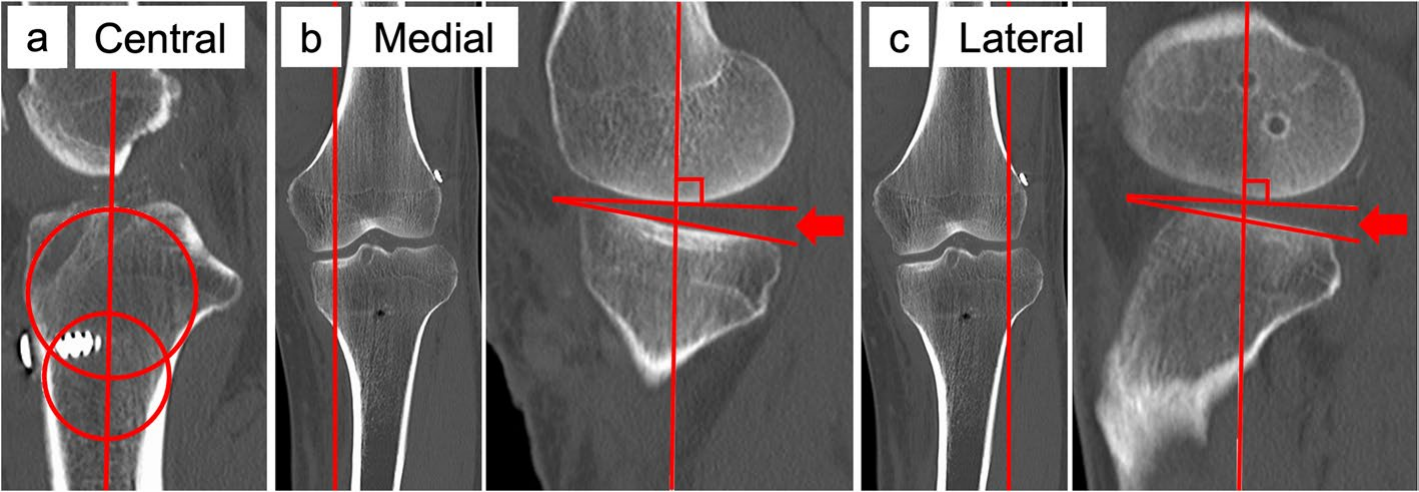
Posterior tibial slope (PTS) measurements using CT images. The arrow indicates the angle of lateral and medial PTS

Medial and lateral PTS (°) was measured respectively as previously reported [14]. Briefly, the central sagittal CT image was selected based on (1) the intercondylar eminence, (2) the anterior and posterior cortices appeared in a concave shape, and (3) the attachment of the posterior cruciate ligament, and tibial axis was determined by connecting the centroids of two circles fitting the tibial shaft (Fig. 2a). A first circle was fitted to the anterior, posterior, and cranial cortexes of proximal tibia, and a second circle was fitted distally tangential to the anterior and posterior cortexes, with its centre placed on the border of the first circle. The centre of the lateral and medial tibial plateau was selected using a coronal CT image, and the medial and lateral PTS were determined by the angle between the axis perpendicular to the tibial axis and the line connecting the two most proximal anterior and posterior subchondral bone surfaces (Fig. 2b, c). The lateral-medial slope asymmetry was calculated by subtracting medial PTS from lateral PTS (lateral-medial PTS). Two examiners (examiner 1 and examiner 2) independently measured lateral and medial PTS to assess the inter-rater reliability. The values of examiner 1 were used for the statistical analysis. Examiner 1 measured lateral and medial PTS twice with eight weeks interval to assess the intra-rater reliability.

### Quantitative evaluation of the pivot-shift test

The pivot-shift test was performed five times prior to the surgeryunder general anaesthesia using the EMS. This system consists of a transmitter that produces an electromagnetic field and three electromagnetic receivers. The two receivers are firmly attached to the thigh and the lower leg, and represent the motion of the femur and tibia, respectively, after setting the coordinate system. Seven anatomic landmarks of lower limbs, including the greater trochanter of the femur, medial femoral epicondyle, lateral femoral epicondyle, the intersection of the medial collateral ligament and knee joint line, fibula head, medial malleoli, and lateral malleoli, were digitized using the third receiver by the surgeon who performed ACL reconstruction, and their three-dimensional positions were recorded relative to the positions of the two receivers on the lower limb. Relative movement between the two receivers during knee motion can be converted to six-degrees-of-freedom knee kinematics in accordance with the Grood and Suntay coordinate system [6]. Tibial acceleration (m/s^2^) during posterior reduction of the tibia was calculated from the data of anteroposterior translation during the pivot-shift test, and the maximum value just before the pivot-shift phenomenon was used for the analysis as previously reported [12, 13, 24]. The average of three medians was used for the analysis.

### Statistical analysis

All statistical analyses were performed using EZR (Saitama Medical Center, Jichi Medical University, Saitama, Japan), which is a graphical user interface for R (The R Foundation for Statistical Computing, Vienna, Austria) [16]. The Shapiro–Wilk test was used for the normality test. All the PTS parameters (lateral PTS, medial PTS, and lateral-medial slope asymmetry) were normally distributed; therefore, Pearson correlation analysis was performed to evaluate the correlation between each PTS parameter (medial PTS, lateral PTS, and lateral-medial slope asymmetry) and tibial acceleration during the pivot-shift test. Statistical significance was set at *p* < 0.05. A priori sample size calculation using G*power 3.1 (Christian Albrecht University, Kiel, Germany) showed that 29 subjects were required to detect a moderate correlation (0.50), assuming a power of 0.80 and an alpha error of 0.05.

Interclass correlation coefficient (ICC) was used to assess inter-rater and intra-rater reliability for lateral and medial PTS. In reference to a previous study, the categorisation of ICC scores was based on the 95% confidence interval (CI) and determined as a priori, whereby ICC < 0.50 indicates poor agreement, 0.50 ≤ ICC < 0.75 indicates moderate agreement, 0.75 ≤ ICC < 0.90 indicates good agreement, and ICC ≥ 0.90 indicates excellent agreement [18].

## Results

**Fig. 3:**
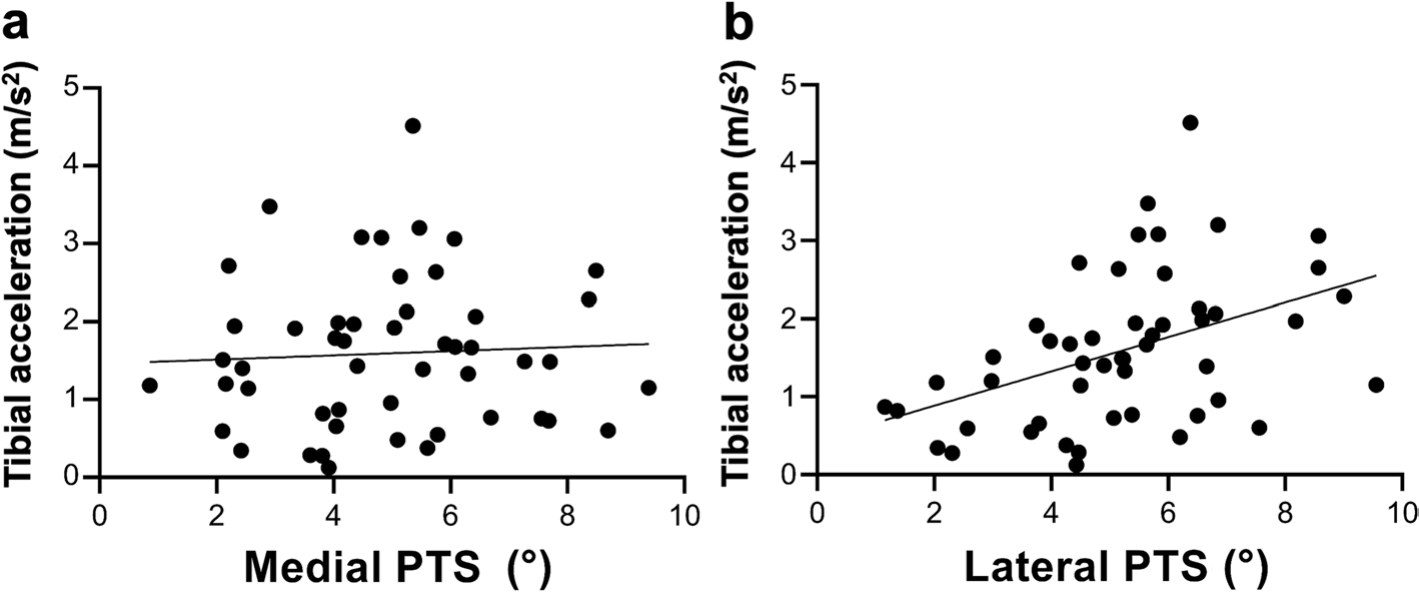
Correlation between posterior tibial slope (PTS) and tibial acceleration during the pivot-shift test. **a** No significant correlation was observed between preoperative tibial acceleration and medial PTS (*r* = 0.06, *p* = 0.70). **b** Tibial acceleration positively correlated with lateral PTS (*r* = 0.436, *p* < 0.01)

**Fig. 4:**
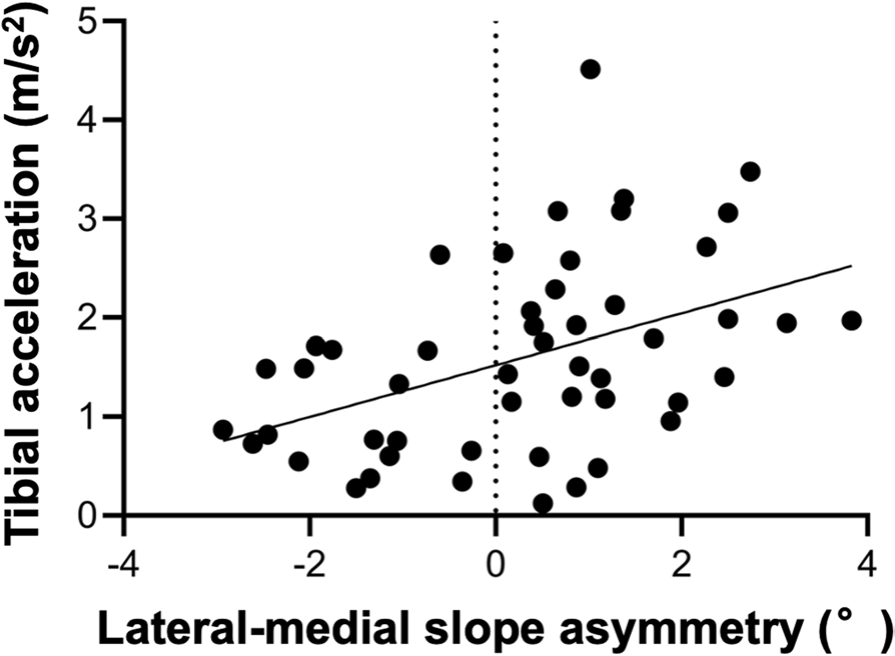
Correlation between lateral-medial slope asymmetry and tibial acceleration during the pivot-shift test. Tibial acceleration positively correlated with lateral-medial slope asymmetry (*r* = 0.443, *p* < 0.01)

The medial PTS was 4.9° (95% CI: 4.3 to 5.5, range: 0.9 to 9.6), and the lateral PTS was 5.2° (95% CI: 4.7 to 5.7, range: 1.2 to 9.6). The inter-rater ICCs of medial and lateral PTS were 0.81 (95% CI: 0.71 to 0.88) and 0.84 (95% CI: 0.76 to 0.90), indicating moderate to good and good to excellent agreement respectively. The intra-rater ICCs of medial and lateral PTS were 0.87 (95% CI: 0.79 to 0.91) and 0.89 (95% CI: 0.82 to 0.93), indicating good to excellent agreement. The lateral-medial slope asymmetry was 0.3° (95% CI: -0.2 to -0.8, range: -2.9 to 3.8). The tibial acceleration during the pivot-shift test in the ACL-injured knee was 1.6 m/s^2^ (95% CI: 1.3 to 1.9, range: 0.1–4.5). While no significant correlation was observed between preoperative tibial acceleration and medial PTS (*r* = 0.06, *p* = 0.70) (Fig. 3a), preoperative tibial acceleration was moderately correlated with lateral PTS (*r* = 0.436, *p* < 0.01) (Fig. 3b), and lateral-medial slope asymmetry (*r* = 0.443, *p* < 0.01) (Fig. 4).

## Discussion

Preoperative tibial acceleration during the pivot-shift test was positively correlated with lateral PTS and lateral-medial slope asymmetry in ACL-injured knees, which supported the hypothesis. This suggests that the magnitude of the preoperative pivot-shift appears to be associated with the bony morphology of the tibial plateau, and especially lateral tibial plateau and the difference in lateral and medial PTS. Thus, surgeons should be aware that not only lateral PTS but also lateral-medial slope asymmetry are the factors associating with preoperative anterolateral rotatory knee laxity in ACL injury. Special attentions will be needed in ACL-injured patients with steep lateral PTS and large lateral-medial PTS asymmetry.

The pivot-shift phenomenon is described as forward subluxation of the lateral tibial plateau on the femoral condyle in extension, and a spontaneous and sudden reduction in flexion. To detect this pivot-shift phenomenon, the pivot-shift test is the most commonly used clinical examination [22]. The preoperative pivot-shift test has particular clinical importance because greater preoperative pivot-shift is shown to be associated with residual post-operative pivot-shift after ACL reconstruction [33]. Moreover, residual post-operative pivot-shift after ACL reconstruction has been shown to associate with symptoms and poor functional outcomes [2], and could be associated with posttraumatic knee osteoarthritis [15]. Therefore, a better understanding of the pivot-shift phenomenon is considered clinically important to prevent the residual post-operative pivot-shift and subsequent complications or worsening of clinical outcomes.

Several factors have been shown to be associated with tibial acceleration during the pivot-shift test, which was measured by the EMS. Nishida et al. reported that chronicity (more than one year after ACL injury) and lateral meniscal injury were associated with increased tibial acceleration during the pivot-shift test in ACL-deficient knees [25], and another study has shown that unrepaired lateral meniscus injury during ACL reconstruction is associated with residual pivot-shift phenomenon one year after surgery [11]. Moreover, previous studies have shown that anterolateral complex injury does not significantly affect anterolateral rotatory laxity in ACL-deficient knees by using the EMS in cadaveric and clinical studies [1, 20]. In the present study, patients with concomitant meniscus injuries were included, however, there was no significant differences in the tibial acceleration between with and without lateral meniscus injuries. Other studies have shown that generalized joint laxity, knee hyperextension, female sex, meniscal tear, anterolateral complex injury, and bony morphology, including small lateral femoral condyle and greater PTS, were the factors associated with greater anterolateral rotatory knee laxity in ACL-deficient knees by using accelerometers or iPad systems, which can also quantify the pivot-shift [23, 26–28, 32]. In addition to these factors, the present study has shown that tibial bony morphology, especially lateral PTS and lateral-medial slope asymmetry, is associated with the magnitude of the preoperative pivot-shift.

Some previous studies have shown that a steeper PTS is a risk factor for a high-grade pivot-shift test [3, 4, 27]. Batty et al. analyzed 618 ACL-injured patients and found that PTS > 9°, measured by lateral plain radiograph, was one of the six factors associated with high-grade pivot-shift [4]. Another study has shown that lateral PTS measured by MRI was significantly greater in a “high-grade rotatory laxity” group (9.3° ± 3.4°) compared to a “low-grade rotatory laxity” group (6.1° ± 3.7°) based on quantitatively assessed pivot-shift test with the image analysis using an iPad [27]. A significant association has also been reported between lateral PTS > 9° and high-grade pivot-shift, and anterolateral ligament injury, while medial PTS was not significantly different between low-grade and high-grade groups [3]. However, as limitations of the previous studies, the association between medial–lateral PTS asymmetry and the pivot-shift was not assessed, and only one study [27] quantitatively assessed pivot-shift test by using iPad image analysis [10] among the three studies [3, 4, 27]. The present study found that not only lateral PTS but also medial–lateral PTS asymmetry were significantly correlated with tibial acceleration during the pivot-shift test, which is a novel finding compared to the previous studies [3, 4, 27].

Greater lateral-medial slope asymmetry indicates that lateral PTS is steeper than medial PTS. Regarding the lateral-medial slope asymmetry, one study proposed a mechanism that an axial loading force would be more likely to cause the lateral side of the femur to slide posteriorly off of the steep lateral tibial plateau, using the flat medial tibial plateau as a pivot point [30], and thus greater pivot-shift phenomenon may occur in the knee with steeper lateral PTS as well as the greater lateral-medial slope asymmetry. The present finding could be supported by this proposed mechanism. One previous study has shown that lateral-medial slope asymmetry was a risk factor for concomitant lateral meniscus posterior root tears in ACL injuries [17], though it did not investigate the relationship with the pivot-shift. Therefore, the present study will add valuable knowledge to the literature.

The present study has some limitations. First, the present study is a retrospective analysis, and the number of subjects was relatively small and the rate of excluded patients was high, which may have caused selection bias. But, a prior power analysis indicated that the current number of subjects was sufficient to detect a moderate correlation. Second, the measurement of PTS was performed by using CT images, and PTS considering the articular cartilage and meniscal slope could not be measured in the present study. However, preoperative MRI scans were often performed at other clinics and hospitals and the quality was not consistent, while CT scans were performed at our hospital and the quality was consistent. Third, the maneuver of the pivot-shift test was performed by several experienced surgeons. A previous study demonstrated that instruction of a standardized pivot-shift test maneuver to multiple examiners could provide a more consistent quantitative evaluation [9], and the influence on the results would have been minimal. Finally, side-to-side difference in tibial acceleration was not determined, which would have been of interest. However, tibial acceleration in the ACL-injured/reconstructed knees have been consistently used in the previous studies, thus we have followed this approach in the present study [1, 8, 12, 20, 24].

## Conclusions

Steeper lateral PTS and greater lateral-medial slope asymmetry were correlated with greater preoperative tibial acceleration during the pivot-shift test in ACL-injured knees. These findings improve our understanding of anterolateral rotatory knee laxity by linking tibial bony morphology to quantitative measurement of pivot-shift phenomenon. Surgeons should be aware that lateral PTS, as well as lateral-medial slope asymmetry, may affect preoperative anterolateral rotatory knee laxity in ACL injury.

## Data Availability

Datasets generated or analyzed during this study are not publicly available due to privacy concerns. However, they are available from the corresponding author upon reasonable request.
